# Pooling skin swabs does not inhibit qPCR detection of amphibian chytrid infection

**DOI:** 10.1371/journal.pone.0214405

**Published:** 2019-04-02

**Authors:** Joana Sabino-Pinto, An Martel, Frank Pasmans, Sebastian Steinfartz, Miguel Vences

**Affiliations:** 1 Zoological Institute, Braunschweig University of Technology, Braunschweig, Germany; 2 Department of Pathology, Bacteriology and Avian Diseases, Faculty of Veterinary Medicine, Ghent University, Merelbeke, Belgium; 3 University of Leipzig, Institute of Biology, Molecular Evolution and Systematics of Animals, Leipzig, Germany; Universitat Trier, GERMANY

## Abstract

Immediate and reliable pathogen detection in large numbers of samples is essential in wildlife disease monitoring and is often realized by DNA-based techniques. Pooling samples increases processing efficiency and reduces processing costs, and has been suggested as a viable technique for quantitative PCR detection of fungal amphibian pathogens of the genus *Batrachochytrium*. For these fungi, this diagnostic method has been validated by *in vitro* set ups that provided controlled test conditions but did not take into account potential effects from amphibian skin compounds (e.g. skin secretions and Microbiota) on the approach. Some of these skin compounds are known to cause PCR inhibition in single sample applications and could lead to false negative reactions and thereby hamper pathogen detection. In this study we examined the effect of skin compounds on the pooled extraction method by swabbing individuals of seven amphibian species (one Anura and six Caudata) prior to the inoculation of the swabs with chytrid zoospores. For each species, swabs were extracted in pools of different sizes (from one to four swabs) with only one swab per pool being inoculated with zoospores. There were no significant differences regarding the ability to detect zoospores when comparing pool sizes for any species, with a tendency for more false negatives when the inoculated swab had been inoculated with a single zoospore. This study provides further *in vivo* evidence for the viability of the pooled extraction method for DNA-based detection of pathogens.

## Introduction

Emerging infectious diseases (EIDs) are diseases that are caused by pathogens that have recently expanded their range, either geographically and/or in host species [1]. They can be divided in three groups: 1) EIDs invading new hosts; 2) EIDs caused by a mutated pathogen (increased pathogenicity in the same host); and 3) EIDs invading new geographical areas (invasive species) [2]. The frequency at which these diseases have been detected has increased over the years [3] and these diseases threaten the health of numerous animals and plants. The control of such infections is of high societal, commercial and political value for animal and human health, food production and safety, and conservation of biodiversity. Many studies focus on hosts or pathogens involved in EIDs, but these studies tend to target humans or livestock pathogens [4–6]; only recently these studies have been extended to wild animals [7]. When tackling EIDs, the first step is the determination of the pathogen distribution, often with molecular (DNA-based) techniques. This requires the collection and processing of large amounts of samples, with time of processing being especially important when sampling for highly infectious and lethal pathogens.

A way to make DNA-based pathogen detection analyses more time and costs-efficient in certain scenarios is by pooling samples at the extraction level. Such an approach does not allow to identify an infection in a specific individual but rather whether within a group of individuals sampled, an infection can be detected; and therefore allows a faster and more cost-effective screening of groups of individuals, e.g. from a certain geographic area for which the main goal is determining presence or absence of a pathogen. The validity of pooling samples prior to DNA extraction followed by a quantitative PCR (qPCR) for pathogen detection has been shown a decade ago for the amphibian fungal pathogen *Batrachochytrium dendrobatidis* (*Bd*) [8], and has been further developed for *B*. *salamandrivorans* (*Bsal*) [9], as a method for reducing work load and costs when screening for this cutaneous pathogen via skin swabs. In both of these studies, sterile swabs were inoculated with different amounts of pathogenic fungi, and the effect of extracting the DNA from several swabs in a single vial is analyzed. The overall conclusion was that the dilution effect originated from pooling samples is negligible and that reliable pathogen detection can be achieved when extracting up to four swabs in a pool. Although these studies validated the pooled extraction method under *in vitro* conditions, the effects of host skin components (e.g. mucus and microbiota) and environmental substances possibly attached to the skin (e.g. soil, water and vegetation) as possible PCR inhibitors was not taken into consideration. These components are not always perfectly removed (and some of them cannot be removed at all) during the cleaning step of the DNA extraction and can interfere with qPCR by directly interacting with the DNA [10], leading to biased results, i.e. potential false negatives.

The two pool extraction studies mentioned [8,9] were developed for the chytrid fungi, *Bd* [11] and *Bsal* [12]. These pathogens cause chytridiomycosis, an amphibian disease that has increased in both range and pathogenicity over the last years, and it has been suggested that chytridiomycosis is contributing to the sixth mass extinction event [13]. The pathogen *Bsal* is of particular concern because it causes rapid declines of fire salamander populations, and is capable of infecting and killing many more European and non-European Caudata [14,15]. Nowadays the invasive range of *Bsal* covers a rough geographic range of 20,000 km^2^ (Wagner; personal communication) within three countries (Belgium, Netherlands and Germany [16]) in Europe and is constantly expanding [17]. An efficient large-scale screening requires a quick flow between sample collection and sample processing; the importance of time is related to the fact that *Bsal* is an extremely deathly pathogen and delay in its detection could move the population over the tipping point towards extinction.

In this study, the performance of the pooled extraction method is tested for *Bsal* while sampling living amphibians by skin swabs. By swabbing the skin of proven *Bsal*-negative individuals prior to the inoculation of the swabs with *Bsal* zoospores we simulate field conditions: our sampling design incorporates the skin components that can act as qPCR inhibitors into the analysis. Seven species of amphibians (one Anura and six Caudata), were swabbed to analyze the validity of the technique in multiple amphibian systems. Swabs were pooled into groups of 1 to 4 swabs, extracted and analyzed by qPCR.

## Material and methods

### Experimental design

All ethical procedures were followed; skin swabs were collected at Ghent University where no permit is required to collect such samples. The non-invasive, non-lethal sampling of amphibians does not cause animal suffering or obvious discomfort and hence does not require prior approval by an ethical committee under the current Belgian legislation with regard to animal experimentation. All sampled individuals are part of the captive collection maintained at Ghent University.

Two experiments were carried out, as detailed below. Experiment 1 was limited to specimens of the fire salamander, *Salamandra salamandra*, and was an *in vivo* replication of the second experiment in Sabino-Pinto et al. [9]. Experiment 2 was a similar to experiment 1 but with only three pool sizes (1, 3 and 4 swabs) and two *Bsal* loads (10 and 1,000 zoospores), performed on six other amphibian species (one Anura–*Alytes obstetricans–*and six Caudata–*Cynops pyrrhogaster*, *Ichthyosaura alpestris*, *Lissotriton helveticus*, *Pachyhynobius shangchengensis*, and *Tylototriton wenxianensis*).

**Fig 1:**
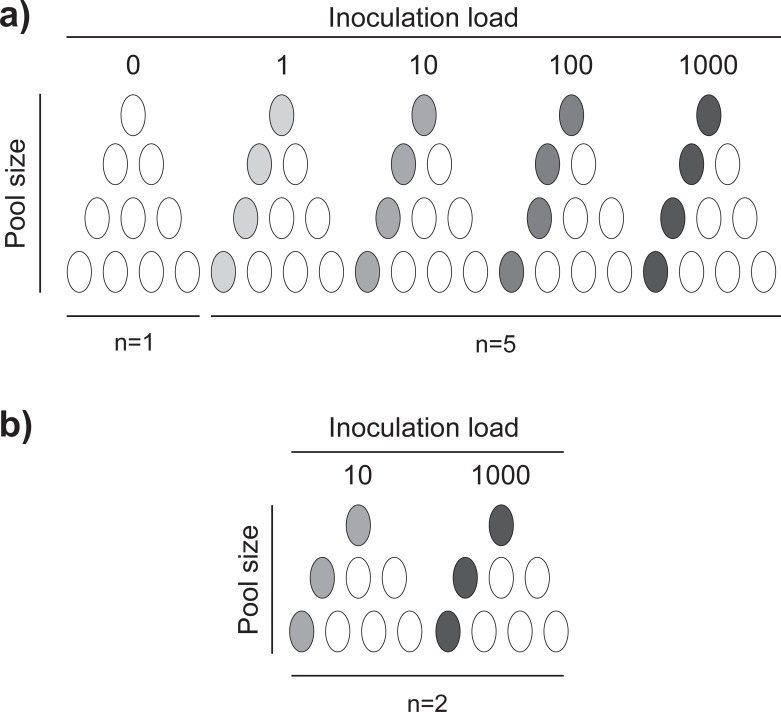
Experimental set up. Experimental set up for pooled extraction of: **a)** Experiment 1, for *Salamandra salamandra*; and **b)** Experiment 2, for *Alytes obstetricans*, *Cynops pyrrhogaster*, *Ichthyosaura alpestris*, *Lissotriton helveticus*, *Pachyhynobius shangchengensis*, and *Tylototriton wenxianensis*. Swabs were pooled in groups of one to four swabs (pool size) with one swab per pool being inoculated with zoospores (inoculation load) (with the exception of the controls). Load in zoospores per swab.

In experiment 1, the validity of the pooled DNA extraction method was determined *in vivo* for *S*. *salamandra*. 200 skin swabs were collected by rubbing the ventral side of captive bred *S*. *salamandra* at Ghent University. All individuals were confirmed to be *Bsal*-free via q-PCR before sample collection. A fraction of the swabs was inoculated with 50 μl of a suspension containing either: 1, 10, 100 or 1,000 *Bsal* zoospores. The remaining swabs were inoculated with 50 μl of sterile water (blanks). The swabs were arranged in groups (hereafter referred to as pools), composed by one swab with zoospores and 0–3 blank swabs (Fig 1A). Each pool size-zoospore load combination was replicated five times. As a control, a replicate of each pool size containing all blank swabs was extracted (Fig 1A); all controls were negative.

In Experiment 2, the pooled DNA extraction method was applied to six other amphibian species. 192 (32 per species) swabs were collected by rubbing the ventral side of captive bred *A*. *obstetricans*, *C*. *pyrrhogaster*, *I*. *alpestris*, *L*. *helveticus*, *P*. *shangchengensis*, or *T*. *wenxianensis* at Ghent University The species were selected based on their availability at the laboratory, on their taxonomy/systematics (Anura and Caudata), and on their sensitivity to chytridiomycosis (susceptible–*S*. *salamandra*, *T*. *wenxianensis*, *C*. *pyrrhogaster* and *I*. *alpestris*; resistant–*A*. *obstetricans*, *L*. *helveticus* and *P*. *shangchengensis* [14]). All individuals were confirmed to be *Bsal*-free via qPCR before sample collection. A fraction of the swabs were inoculated with 50 μl of suspension containing either: 10 or 1,000 *Bsal* zoospores. The remaining swabs were inoculated with 50 μl of sterile water (blanks). The swabs were arranged in groups, composed by one swab with zoospores and 0, 2 or 3 blank swabs (Fig 1B). Each pool size-zoospore load combination was replicated two times (Fig 1B).

### *Bsal* culture and harvest

Inoculation of swabs was done with zoospores of the *Bsal* isolate AMFP13/1, collected from a fire salamander (*Salamandra salamandra*) in the Netherlands. Cultures of this isolate were grown in 25cm^3^ cell culture flasks in TGhL broth and incubated at 15°C for five days. After the incubation, cultures were supplemented with mPmTG/sterile deionized water and incubated overnight at 15°C to stimulate zoospore release. Stimulated cultures were filtered with a 20 μm filter to remove zoosporangia. Zoospore concentrations were determined with a hemocytometer with light microscopy using five replicate counts.

### Sample processing

Total DNA was extracted from the pooled swabs with the Qiagen DNeasy Blood and Tissue Kit (Qiagen, Hilden, Germany) following the Animal Tissues protocol with the pre-treatment for gram-positive bacteria and expanding the initial incubation to 1 h, as well as carrying out the second incubation at 70°C [18].

The qPCR assays amplified a fragment of the (ITS)-5.8S rRNA region following the simplex method of Blooi et al. [19] using KlearKall Master Mix (LGC group, Middlesex, UK) instead of Taqman Universal Master Mix (1xiQ Supermix; Bio-Rad Laboratories, Hercules, CA). qPCRs were performed on a CFX96 Real-Time System (Bio-Rad Laboratories Inc., Hercules, CA). Samples were run in duplicate with a third replicate being run when original duplicates were not in agreement. All qPCR plates had a negative control and a set of four standards (i.e. 0.1, 1, 10, 100, 1,000 zoospores), all in duplicate.

### Data analysis

Samples were considered positive when two of the qPCR replicates had an amplification signal between the lowest and highest standards, the amplification curve was logarithmic, and the standard error was smaller than the mean. In cases, in which duplicates showed contradictory results, a third replicate was run to break the tie. The amount of zoospores present in a sample (i.e. zoospore equivalents) was determined as the average of the replicates for that sample, excluding the negative replicate in the cases with a third replicate. To account for the extraction volume, the average values were then multiplied by 10. Raw data can be found in S1 Table.

Differences in efficiency (i.e. the ability to detect a *Bsal* signal; binomial response: positive/negative) between pool sizes for *S*. *salamandra* (experiment 1) and other species (experiment 2) were calculated by categorizing the data into presence-absence. This data was then fitted to a generalized linear model (GLM) with the assumption of a binomial distribution with pool size, load and their interaction as factors.

Differences in load counts (i.e. detected *Bsal* zoospores per swab) were analysed by fitting the data to a linear model (LM) with pool size, load, and their interaction as factors. Shapiro-Wilk tests were used to control for the normal distribution of the residuals, and Levenes tests for the homogeneity of variance. If the residuals were found to be not normally distributed, the detected zoospore numbers were log (x) transformed. Linear models were backward selected,–i.e. if the interaction was not significant it was excluded, while main factors always remained.

Samples with 1,000 zoospores with a pool size of one that did not amplify had 10 extra replicates collected and processed; this way we excluded potential human issues not associated with the method itself. This was done for *I*. *alpestris*, *P*. *shangchengensis*, or *T*. *wenxianensis*.

All tests were calculated on R 3.3.1 [20] and the package car [21].

## Results

### Experiment 1 –*S*. *salamandra*

In this experiment, chytrid free *S*. *salamandra* individuals were swabbed, the swabs were subsequently inoculated with pre-determined amounts of zoospores or sterile water, and pooled with blank swabs (0 to 3) for extraction (i.e. all swabs from a specific pool were placed in a vial and extracted as one).

The size of the pool had no direct effect on the detectability (presence/absence) of the *Bsal*–fungus (GLM: χ^2^_(1,3)_ = 0.14, p-value = 0.704; Table 1), or on the amount of *Bsal* zoospores detected (LM: F_(1,71)_ = 2.10, p-value = 0.152; Table 1).

**Table 1:** Zoospore counts for Experiment 1.

Load of the inoculated swab		1 zoospore			10 zoospores			100 zoospores			1,000 zoospores	
Pool size	O/E	Load	Std	O/E	Load	Std	O/E	Load	Std	O/E	Load	Std
**1**	**4/5**	12.62	10.3	5/5	38.99	33.7	5/5	924.33	636.3	5/5	2,771.24	3,100.3
**2**	**3/5**	26.36	2.4	5/5	44.97	55.8	5/5	767.65	522.0	5/5	1,453.58	1,412.6
**3**	**2/5**	3.05	1.9	**4/5**	10.72	6.3	5/5	629.41	459.5	5/5	1,697.45	1,398.7
**4**	**4/5**	8.71	5.7	5/5	32.24	37.9	5/5	334.62	299.2	5/5	2,320.15	2,764.5

Detectability and zoospore amounts of the detected loads of *Batrachochytrium salamandrivorans* for *Salamandra salamandra* according to the pool size, and load of the inoculated swab. O/E: Number of pools with positive signal for the chytrid fungi and the total number of pools processed. Load: average number of zoospores per pool estimated from the qPCR signal. Std: Standard deviation. Bold values indicate groups in which not all samples amplified.

Approximately half of the pools containing a swab with only one theoretical zoospore (7/20 replicates), across all pool sizes, did not amplify (1, 2, 3, 1 samples in pool sizes 1, 2, 3, 4, respectively). Additionally, one out of 20 pools containing a swab inoculated with 10 zoospores did not amplify (pool size = 3) (Table 1).

### Experiment 2 –Other species

The same pool approach as in experiment 1, with pool sizes of 1, 3 and 4 and inoculation loads of 10 and 1,000 was applied to six other amphibian species.

The size of the pool and the species had no direct effect on the detectability (presence/absence) of the fungi for any of the species (GLM: pool– χ^2^_(1,8)_ = 0.02, p-value = 0.881; species– χ^2^_(5,8)_ = 1.07, p-value = 0.957). The interaction between pool size and species was considered statistically significant (LM: pool*species–F_(5,81)_ = 6.17, p-value < 0.001). There was an effect of species on the detected loads, which was not linked to pool size (LM: species–F_(5,81)_ = 4.00, p-value = 0.003; pool–F_(1,81)_ = 25.24, p-value < 0.001).

Across all species, loads and pool sizes, seven samples did not amplify: one sample from *A*. *obstetricans* (pool size = 4, inoculation load = 10 zoospores), three from *I*. *alpestris* (pool size/inoculation load: 1/1,000, 3/10 and 4/10), one from *L*. *helveticus* (pool size = 4, inoculation load = 10 zoospores), one from *P*. *shangchengensis* (pool size = 1, inoculation load = 1,000 zoospores), and one from *T*. *wenxianensis* samples (pool size = 1, inoculation load = 1,000 zoospores) (Table 2).

**Table 2:** Zoospore counts for Experiment 2.

Pool size			1						3						4			
Load of the inoculated swab		10			1,000			10			1,000			10			1,000	
	O/E	Load	Std	O/E	Load	Std	O/E	Load	Std	O/E	Load	Std	O/E	Load	Std	O/E	Load	Std
*A*. *obstetricans*	2/2	116.93	60.02	2/2	3,246.18	603.49	2/2	20.10	17.10	2/2	106.37	84.14	**1/2**	47.15	NA	1/1	174.46	NA
*C*. *pyrrhogaster*	2/2	6.72	1.81	2/2	3,346.99	3,295.25	2/2	38.39	28.78	2/2	1,205.11	719.35	2/2	42.81	26.76	2/2	4,607.03	3,941.25
*I*. *alpestris*	2/2	76.56	52.62	**11/12**	1,599.27	787.18	**1/2**	27.07	NA	2/2	143.32	93.33	**1/2**	37.75	NA	2/2	114.21	110.57
*L*. *helveticus*	2/2	117.66	37.68	2/2	3,778.01	3,511.48	2/2	9.06	3.76	2/2	6,128.33	278.52	**1/2**	28.90	NA	2/2	1,031.85	533.76
*P*. *shangchengensis*	2/2	39.94	10.28	**11/12**	1,219.64	350.52	2/2	33.47	27.62	2/2	7,750.16	4,177.92	2/2	86.97	50.20	2/2	846.36	64.46
*T*. *wenxianensis*	2/2	234.77	16.44	**11/12**	1,520.55	335.18	2/2	82.10	38.92	2/2	5,045.95	3,337.22	2/2	389.91	301.46	2/2	5,103.21	2,479.28

Detectability and zoospore amounts of the detected loads of *Batrachochytrium salamandrivorans* for *Alytes obstetricans*, *Cynops pyrrhogaster*, *Ichthyosaura alpestris*, *Lissotriton helveticus*, *Pachyhynobius shangchengensis*, *Tylototriton wenxianensis* according to the pool size, and load of the inoculated swab. O/E: Number of pools with positive signal for the chytrid fungi and the total number of pools processed. Load: average number of zoospores per pool estimated from the qPCR signal. Std: Standard deviation. Bold values indicate groups in which not all samples amplified.

## Discussion

This study revealed that *in vivo* conditions of cutaneous swabbing of amphibians do not influence the ability to detect the presence of a fungal pathogen DNA by qPCR after a pooled DNA extraction. This conclusion applies to various amphibian species differing in their susceptibility to *Bsal*: the hyper-susceptible *S*. *salamandra* and *T*. *wenxianensis*, the moderately susceptible *C*. *pyrrhogaster* and *I*. *alpestris*, and the seemingly resistant *A*. *obstetricans*, *L*. *helveticus* and *P*. *shangchengensis* [14].

Samples from all pool sizes (1–4) and with all inoculation loads (1–1,000) consistently showed amplification signals (Tables 1 and 2). There were no significant differences between pool sizes and detected loads; therefore the pooled DNA method can be seen as a valid method to increase efficiency when processing large numbers of samples for the genetic detection of *Bsal* in amphibians. While amplification signals were consistently found, the number of zoospores detected (calculated via qPCR) often did not correspond to the exact inoculated number. These variations in load counts from the inoculated values to the qPCR determined ones are likely related to the fact that chytrid cultures that have been kept active for several passages change drastically the chromosomal copy number, and therefore the ITS copy number (the fragment that is being amplified) [22–24]. Consequently, as one possible explanation for our high load estimates, the culture used to develop the qPCR primers may have had less ITS copies than the one used to inoculate the swabs.

As recommended previously [9], the suggested pooled extraction method should be combined with simultaneous collection of duplicate samples. With this approach, the pool analysis can be used to determine if a pathogen is detected in groups of samples (pools). Subsequently, if needed, the duplicate samples from the samples in those pools can be individually extracted to confirm infection of specific individuals.

The potential of false negatives calls for caution when using the pooled approach. This pooled DNA extraction method is reliable for large monitoring and screenings where the presence and absence of a pathogen (e.g. *Bsal*) is to be verified. In such situations, false positives might therefore be less a concern, especially if a duplicate swab is available for confirmation. However, this approach is not recommended in cases where specific individuals are investigated for the occurrence of a pathogen; here, false negatives can have drastic repercussions, such as screening of quarantine individuals and individuals for possible translocation.

This study has extended the applicability and validity of the pooled DNA extraction method to the situation of the emerging pathogen infection *Bsal* that is currently spreading its geographic and host range in Europe. In the right context, this approach can accelerate the pathogen screening process and make it more cost effective.

## Supporting information

S1 Table Raw data. (XLSX)
